# Improved medical protective clothing comfort for medical staff facing high-intensity infectious diseases

**DOI:** 10.3389/fpubh.2025.1643043

**Published:** 2025-08-11

**Authors:** Cheng-teng Jiang, Dong-yu Chen, Rong Zhang, Fei Ren, Han-wen Zheng, Yuan Yue, Shi-quan Xing, Chuang Nie, Jian-wen Gu

**Affiliations:** ^1^The Ninth Medical Center of Chinese PLA General Hospital, Beijing, China; ^2^Medical School, University of Chinese Academy of Sciences, Beijing, China; ^3^Strategic Support Force Xingcheng Special Duty Sanatorium, Huludao, China

**Keywords:** medical protective clothing, comfort, new integrated design, physiological test, convenience

## Abstract

**Introduction:**

During outbreaks of respiratory infectious diseases such as the COVID-19 pandemic, healthcare workers are frequently required to wear medical protective clothing (MPC) for prolonged periods. Traditional MPC, however, prioritizes protective efficacy while neglecting wearer comfort. Its poor air permeability can induce physical fatigue and dehydration in medical staff during extended use, thereby exacerbating their burden when managing severe infectious diseases. Accordingly, this study aims to enhance the comfort of existing MPC, which holds significant implications for improving the working conditions of medical staff in protracted infectious disease scenarios.

**Methods:**

To address this challenge, a novel integrated MPC was developed through the recombination of mainstream materials. A total of 208 volunteers participated in the experiment, yielding 196 valid data points (81 males and 115 females).

**Results:**

Comparative assessments revealed that the new MPC enhanced air permeability to 120 mm/s, reduced sweating by 30%, decreased skin temperature by 1.2°C, and lowered the incidence of skin maceration by 45%. Furthermore, it shortened donning and doffing time from 5 to 3 minutes.

**Discussion:**

These findings offer a practical strategy for improving MPC comfort without compromising its protective capabilities.

## Introduction

The prevention of infectious diseases has always been a paramount concern in public health (1). During the 2019 COVID-19 pandemic (2, 3), providing precise and effective protection for medical personnel is an important measure for the prevention and control of infectious diseases (4). Medical protective clothing (MPC) is a crucial piece of protective equipment that ensures the safety, well-being, and productivity of medical personnel (5).

Currently, the widely used built-up MPC is complex and cumbersome (6), leading to discomfort and inconvenience (7). The process of donning and doffing the built-up MPC involves up to ten steps including wearing hats, masks, eye masks, goggles, protective clothing, and face screens. The entire process can take approximately 10 to 20 min. Except for positive pressure ventilation suits, all current MPCs have assembled structures that necessitate additional masks and goggles for adequate protection. Consequently, air leakage between the mask and the face can cause fogging in the goggles. Moreover, the inevitable gaps between the assembled components of MPC objectively increase the possibility of infection (8). Medical personnel must repeatedly check for skin exposure between masks, goggles, and protective clothing due to this concern. As a result, this fear leads medical personnel to constantly ensure proper tightness in wearing masks and goggles while also utilizing tape to seal the gap between protective clothing and the body. However, these remedies compromise the breathability of the existing MPC.

In addition, the current use of MPC is associated with inadequate air and moisture permeability (9, 10), cumbersome wearing and removal procedures (11), and the risk of facial compression injuries. These factors significantly impede work efficiency and even pose potential health risks to frontline clinical personnel (6, 12). During the SARS epidemic, frontline medical workers were looking forward to an MPC that was both heat resistant and economical (13). Although the performance of MPC in China is constantly improving, it still cannot meet the requirements of convenience and comfort for frontline medical personnel. Moreover, the utilization of MPC is becoming increasingly prevalent in the present day, thereby creating medical protective clothing possessing superior air and moisture permeability has become more urgent (1, 7).

This study aims to design a one - piece, coverall MPC with a head cover. By integrating the screen, breathing components, and head cover and using a more breathable and moisture - permeable fabric, we strive to enhance the protective efficacy of the MPC while improving wearing comfort. The integrated design can effectively reduce the number of connection points, thereby minimizing the risk of air leakage and infection. Additionally, improving breathability is essential to address the discomfort caused by excessive sweating and heat accumulation, ultimately enhancing the working experience and efficiency of medical personnel.

## Methods and materials

### Material and measures

**Figure 1:**
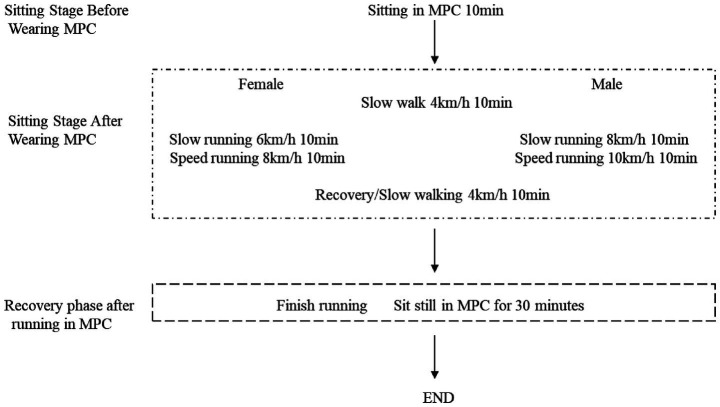
Procedure. Each time, the participants wore different brands of protective clothing and underwent a 10-min period of rest to stabilize their heart rate and allow their bodies to acclimate to the protective gear. Subsequently, their pre-exercise measurements including heart rate, blood oxygen saturation, blood pressure, and blood glucose levels were recorded. Following this, both male and female subjects engaged in a 10-min warm-up session on the treadmill at an initial speed of 4 km/h while wearing the protective clothing to simulate slow movement conditions. Afterwards, female jogged for 10 min at a speed of 6 km/h while male jogged at a speed of 8 km/h to replicate slow exercise scenarios with protective clothing. This was followed by another phase where females ran slightly faster for 10 min at a speed of 8 km/h and males ran at a speed of 10 km/h to mimic faster exercise situations while wearing the protective gear. Finally, all participants resumed walking on the treadmill at a speed of 4 km/h for an additional 10 min to simulate post-exercise recovery after more intense physical activity while wearing the protective clothing. Subsequently, they wore the respective brands of protective clothing for an extended duration of 30 min in order to assess their effects on recovery following relatively long-distance exercise.

Wireless electronic thermometer (Shua Xin, China, RIT-P02-MED) - The body temperature of the subjects was collected before wearing three different brands of protective clothing according to the process in Figure 1, after sitting for 10 min, after exercise, and half an hour after exercise. Libre flash glucose monitoring system (Abbott Diabetes Care Ltd. England, 6,752,835-OMOO683GARR) - It was worn on the upper arm 5 cm near the underarm of the subject, and used to collect the blood glucose changes of the subjects before wearing three different brands of protective clothing, after wearing it for 10 min, after exercise, and after exercise for 30 min in the process of Figure 1. Disposable peripheral blood sampler (Sterilrnce, China), blood glucose test strip (Roche, Switzerland) - It was used to collect the fasting blood glucose values of the subjects for 8 h and more, and to calibrate the initial value of the blood glucose system for each subject. GSP temperature (humidity) recorder (Elitech Technology, China, GSP-8A) - It was used to measure the temperature and humidity of the experimental environment so as to be consistent with the conditions in the hospital ward during the winter of the experiment. Omron electronic blood pressure monitor (Omron Corporation, Japen, U726L) - It was used to collect the blood pressure of the subjects after 30 min of exercise before wearing three different brands of protective clothing in Figure 1. Wrist ECG blood pressure recorder (Huawei, China, MLY-B10) - Used to collect the blood pressure of the subjects after exercise after wearing three different brands of protective clothing for 10 min in Figure 1. Medical disposable protective clothing (DuPont™ Tyvek® 600, America, TY0198TWHEC) - As an international protective clothing brand for the control group. Medical disposable protective clothing (Donbei, China, No. Luxi standard 20,202,140,108) - As China’s general control group protective clothing brand.

Wechsler Adult Intelligence-Memory Scale V4.0 part of the foundation (14, 15). It was used to evaluate the basic logical thinking, calculation and analysis ability of the subjects after 30 min of exercise wearing three different brands of protective clothing in Figure 1. The Personal Thermal Sensation Rating Scale and the Personal Acceptance Statement Scale (Scale were translated from the ISO 10551 Ergonomics of the Physical Environment (16). Subjective judgement scales for assessing physical environments; Gunnar A. V. Brog’s 15-grade scale for ratings of perceived exertion, the RPE Scale, was used to assess the subjective sensory fatigue scale (17–20). Used to evaluate the subjective heat sensation, fatigue, and acceptance of the brand after 30 min of exercise wearing three different brands of protective clothing in Figure 1.

### Procedure

In order to simulate the physiological state of the human body during real medical assistance with various brands of protective clothing. We designed a speed-varying treadmill exercise for men and women. That also allows us to assess the impact of different designs on physiological comfort and ease of donning and doffing (Figure 1).

To simulate the physiological state of the human body during real medical assistance when wearing various brands of protective clothing, we designed a speed-varying treadmill exercise for men and women. A total of 208 volunteers participated in the experiment, and the inclusion criteria were that they were engaged in medical and related industries, aged 25–45 (the main reason is that this age group constitutes the majority of frontline medical staff engaged in high-intensity infectious disease prevention and control work), and had good physical and mental health. A total of 196 effective data points were collected (81 male and 115 female). The volunteers were divided into three groups the random number method. The volunteers wore the same operating clothes inside, and each group was required to wear the corresponding brand of MPC to participate in various tests. The three groups were compared and tested for self-developed and DuPont protective clothing (33 in total, 9 male and 24 female), self-developed and Dongbei protective clothing (41 in total, 20 male and 21 female), and self-developed, DuPont and Dongbei protective clothing (123 in total, 52 male and 71 female). The same volunteer rested for more than six hours before testing the second suit (Figure 1).

The environmental conditions in the ward were simulated, including ambient temperature of 23 ± 1°C and relative humidity ranging from 50 to 60% (9, 21). The experiment recorded the Duration of wearing and taking off protective clothing, as well as the weight of both protective clothing and hand-washing clothes before and after the experiment. Three specific moments were selected for analysis: sitting before any physical exertion, 20 min after dressing, and 30 min after running. Various indicators, such as heart rate, blood oxygen levels, blood glucose levels, blood pressure, body temperature, and short-term working memory, were measured at each designated time point. This study was reviewed by the Ethics Committee of the PLA Strategic Support Force Characteristic Medical Center and conducted in accordance with the Declaration of Helsinki.

### Statistical analysis

Statistical analyses were performed using SPSS23 (SPSS, Inc., Chicago, IL, USA), Graphpad Prism9.0 (GraphPad Software, USA). Two-Way ANOVA analysis of variance was used for comparison between groups. In the analysis of comparisons within groups, when the variance of measurement data was homogeneous and normally distributed, a t-test was used; otherwise, the Mann–Whitney U test was used. The Chi-square test or non-parametric test was carried out for counting data comparison. Statistical significance was set at *P < 0.05, **P < 0.01, ***P < 0.001.

## Results

### New design in protective medical clothing

#### Constructure

**Figure 2:**
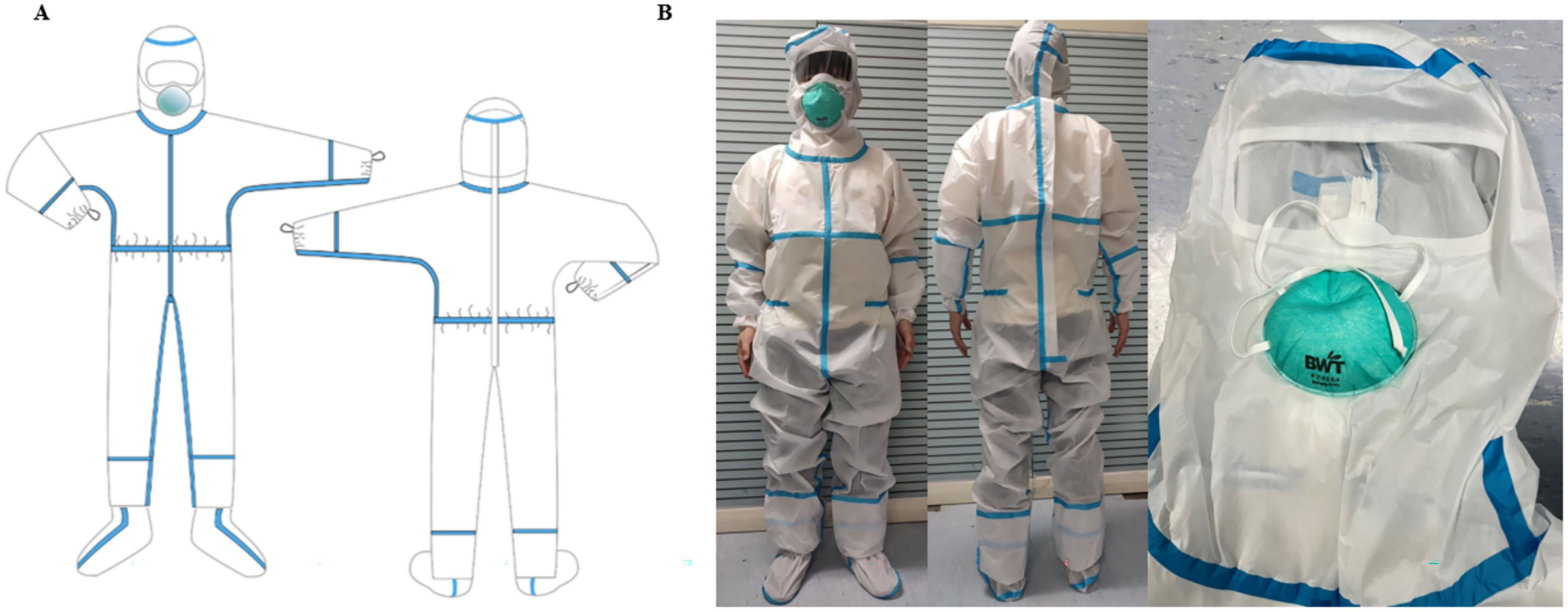
Constructure of medical protective clothing. **(A)** Structure of self-developed MPC; **(B)** Physical sample of self-developed MPC.

MPC adopts a completely enclosed one-piece design. The cuff was fitted with an elastic band, and a structure consisting of inner and outer cuffs was designed. An outer cuff was used to cover the seam of the glove to provide better protection. The cuff is also designed with a finger loop structure, which can effectively fix the protective clothing cuff and prevent it from slipping upward. In addition to the double-layer (22) pant structure, the pant legs were also connected to the socks to better protect the connection between the boots and clothing. A zipper structure was designed on the back, and the blue strip part of the back is shown in Figures 2A,B, which realizes the convenience of donning and doffing and ensures the protective effect of the front of the MPC. The waist circumference of the protective suit can be adjusted to meet the needs of different body types and improve fitness (Figures 2A,B). To increase the effect of facial protection, the original structure, where masks, goggles, and protective clothing were separated, was changed to an enclosed sealing structure. The fabric was seamlessly fused with the mask using ultrasound, ensuring comprehensive facial protection through the addition of a large eye mask (Figures 2A,B). The traditional chest zipper design was replaced with a back zipper design simultaneously, aiming to minimize the risk of contamination on the chest area.

#### Potential leakage points

In terms of protective efficacy, the self-developed MPC, with its fully enclosed one-piece design, achieves a significant reduction in potential leakage points (3 ± 1) compared to the 8 ± 2 in traditional MPCs (DuPont and Dongbei MPC), thereby lowering infection risks. This improvement stems from comprehensive structural optimizations: its integrated facial sealing structure, formed by ultrasonic seamless fusion of fabric and mask along with a large eye mask, avoids the gaps between separate masks, goggles, and clothing that are common in DuPont and Dongbei MPCs; the inner-outer cuff structure with finger loops prevents upward slippage and covers glove seams, addressing the loose or ill-fitting cuffs of traditional MPCs that often form gaps; the double-layer pant legs connected to socks eliminates the separation between pant legs and boots, a frequent leakage source in DuPont and Dongbei designs; and the back zipper (replacing traditional chest zippers) and adjustable waist enhance fit, reducing gaps at the chest and waist typical in conventional MPCs. In contrast, DuPont and Dongbei MPCs, relying on split components with numerous seams and less secure closures, exhibit more leakage points at interfaces like mask-clothing junctions, cuffs, pant-boot connections, and chest zippers, resulting in their higher number of potential leakage points.

#### Base fabric

Through a comparative test of different fabrics, it was found that the polyester material can adsorb ethylene oxide well, but ethylene oxide is difficult to dissociate after the elimination of the fabric; therefore, the polypropylene non-woven fabric is preferred as the base fabric layer of MPC. In medical settings, static electricity can lead to a series of problems. It is important to enhance the antistatic properties of MPC materials, it may attract dust and particles, which could contaminate the sterile environment. Moreover, it can cause interference with sensitive medical equipment, affecting accurate diagnosis and treatment. By enhancing the fabric’s antistatic performance and electrostatic dissipation speed, we aim to create a safer and more stable working environment for medical staff and patients (23). On this basis, a polypropylene nonwoven fabric was further developed to improve its antistatic performance and electrostatic dissipation speed. The overall antistatic performance of the fabric was optimized, and the final overall electric charge was 0.3μC for every piece, as shown in Tables 1, 2.

**Table 1:** Ethylene oxide content of polyester composite fabric under different analytical conditions.

Analytical conditions	Forced dissociation 96 h	Forced dissociation 120 h	Forced dissociation 264 h	Forced dissociation 48 h + natural dissociation 216 h
Ethylene oxide content (μg/g)	80.7	34	135.1	111.1

**Table 2:** The residual amount of ethylene oxide after 48 h analysis of different fabrics.

Fabric type	Dissociation conditions	Ethylene oxide content (μg/g)
Terylene	Natural dissociation 48 h	26.92
Terylene	Forced dissociation 48 h	20.16
Polypropylene fiber	Forced dissociation 48 h	8.72

#### Barrier film

The designed MPC adopts a waterproof and moisture-permeable microporous breathable film as the main protective layer (24), because this material is not only thin and has a strong protective ability, but also has high breathable and moisture-permeable performance, so it can effectively reduce the fabric’s wet resistance and improve the comfort of wearing. The film has good chemical stability because no chemical groups can react with ethylene oxide, and the resolution of ethylene oxide is rapid after its elimination. A microporous permeable film can effectively block particulate matter (diameter 0.075 μm ± 0.020 μm, Test according to GB19082-2009, China) while maintaining good permeability and moisture permeability. The microporous breathable film was biaxially stretched to form nanoscale micropores, which allowed air to pass through but blocked fine solid particles to achieve a particle barrier effect. In addition, by changing the film stretch ratio, the permeability of the film can be improved without compromising its thickness, thus achieving a balance between comfort and durability.

#### Composite technology

Adopt a composite process that considers both air and moisture permeability and scratch resistance. Owing to the conflict between the fabric’s air and moisture permeability and scratch resistance, the two properties were further balanced by improving the film thickness and aperture. At the same time, composite processes such as solvent adhesives (25), melt adhesives, and hot pressing composites have been tested to solve the problem of the low fastness of fabric stripping.

The hot-pressing composite process was used to solve the problem of plugging the pores of the microporous permeable film and reducing moisture permeability and air permeability. Moreover, the hot-pressing composite process causes the film to preshrink to a certain extent, resulting in a small reduction in the number of micropores and improved filtration efficiency, film thickness, and durability. By using the composite substrate and film, the two aspects were fully integrated, resulting in reduced differences in tensile ratios and increased resistance to damage during use. Additionally, the air permeability, moisture permeability, and durability were balanced to achieve optimal performance in both aspects. After testing, the air permeability of the fabric reached 23.1 mm/s or higher, and the moisture permeability reached 6,690 g/ (m^2^·24h).

#### Overall performance of protective clothing

The basic performance of disposable medical protective clothing designed by three different brands was summarized. It was found that the self-made protective clothing had better air permeability in the clothing, and also showed better fabric toughness in the vertical and horizontal tensile tests, and the key indicator of anti-synthetic blood penetration test results were the highest, indicating that compared with the other two medical protective clothing more commonly used during the COVID-19 epidemic, The self-made medical protective clothing is qualified in the microbial detection of the basic performance indicators compared with the other two kinds. In the Efficiency of filtration, Surface resistance to moisture, Moisture permeability, Strength of break, Elongation at break, Rate of air permeability and other indicators of self-made protective clothing are better than the other two brands of protective clothing. According to the test results of the materials, the self-made medical protective clothing is better in the toughness and comfort of the basic clothing (Table 3) (The self-developed protective clothing results were sent to TianFangBiao Standardization Certification & Testing Co., Ltd. National Clothing Quality Inspection and Supervision Center (Tianjin China), National knitted Product Quality Inspection and Supervision Center (China); DuPontTM Tyvek® 600 TY0198TWHEC, From DuPont China web product performance description, https://www.DuPont.cn/products/DuPont-tyvek-600-ty198t-wh.html. Dongbei, the disposable medical protective clothing from Dongbei is registered under the whole code No. Luxi standard 20,202,140,108, which describes the performance indicators of product packaging).

**Table 3:** Basic performance of self-developed, DuPont and Dongbei protective clothing.

Items of inspection	Standard value	Self-developed measured value	DuPont measured value	Dongbei measured value	Standards
Microbial indicators	Total fungal colonies ≤100 CFU/g Total bacterial colonies ≤200 CFU/g Related pathogenic bacteria should not be detected	Pass	Pass	Pass	GB/T 14233.2–2005
Antistatic property	≤ 0.6 uC/Garmalets	≤ 0.3 uC/Garmalets	Pass	Pass	GB/T 12703–91
Efficiency of filtration	> 70%	≥ 99.31%	Pass	Pass	GB 19082–2009
Strength of break	Longitudinal direction > 45 N Transverse direction > 45 N	Longitudinal direction ≥ 124 N Transverse direction ≥ 62 N	Pass	None	GB/T 3923.1–1997
Elongation at break	Longitudinal direction > 15% Transverse direction > 15%	Longitudinal direction ≥ 64% Transverse direction ≥ 59%	None	Pass	GB/T 3923.1–1997
Surface resistance to moisture	> Class 3	4/5 Class	3/5 Class	3/5 Class	GB/T 4745–1997
Water permeability resistance	> 1.67 kPa (17 cm H_2_O)	6.22 kPa	Pass	Pass	GB/T 4744–1997
Rate of air permeability	/	23.1 mm/s	None	None	GB/T 5453–1997
Moisture permeability	> 2,500 g/(m^2^·d)	≥ 6,690 g/(m^2^·d)	Pass	Pass	GB/T 12704–1991
Resistance to synthetic blood penetration	> 1.75 kPa	6/6 Class	3/6 Class	3/6 Class	GB 19082–2009
Ethylene oxide content	< 10 ug/g	< 2 ug/g	Pass	Pass	GB/T 14233.1–2008
Mask filtration efficiency (1 class)	≥ 95%	≥ 99.33%	None	None	GB 19083–2010
Mask airflow resistance	≤ 343.2 Pa	≤ 204 Pa	None	None	GB 19083–2010
Mask surface resistance to moisture	≥ 3 Class	3 Class	None	None	GB/T 4745–1997
Transmittance of protective face screen	/	90.4%	None	None	GB/T 2410–2008

### Variation of donning and doffing

**Figure 3:**
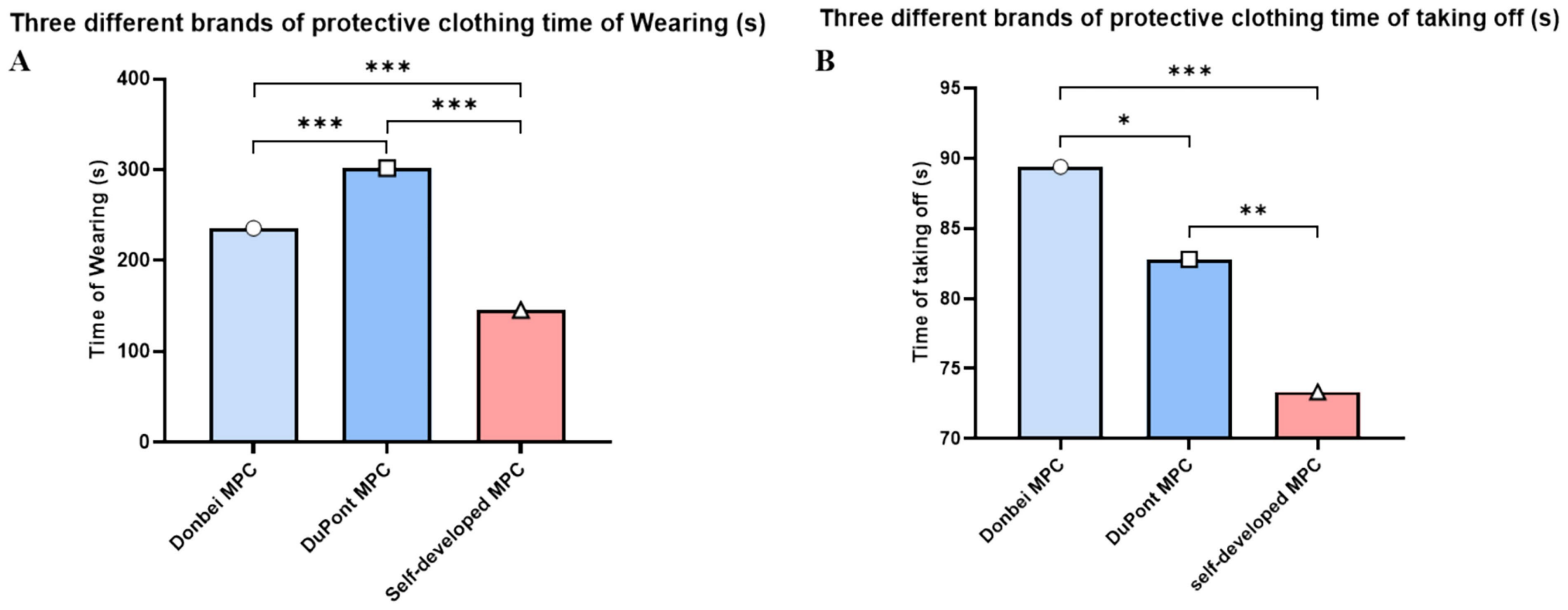
Variation of donning and doffing. **(A)** Time spent donning different MPC; **(B)** Time spent doffing different MPC. (*p ≤ 0.05, **p ≤ 0.01, ***p ≤ 0.001) (two-way ANOVA with post-hoc tests).

The MPC donning and doffing tests involved 20 participants. The duration of each participant was also analyzed. As shown in Figure 3A, Dongbei (China), DuPont (U. S. A) and self-developed MPC had significant differences in dressing time between groups (Dongbei; DuPont; self-developed; 235.8 ± 7.8 s; 301.8 ± 5.0 s; 145.8 ± 11.0 s). The self-developed group had the least dressing time, while the DuPont group had the most dressing time.

In terms of doffing time, there were significant differences among the three MPC groups. It took the least time to develop self-development, followed by DuPont, and the Dongbei Formation, as shown in Figure 3B. (Dongbei; DuPont; self-made; 89.4 ± 4.5 s; 82.8 ± 6.1 s; 73.3 ± 4.9 s), Weight difference between protective clothing and operating clothes before and after exercise.

**Figure 4:**
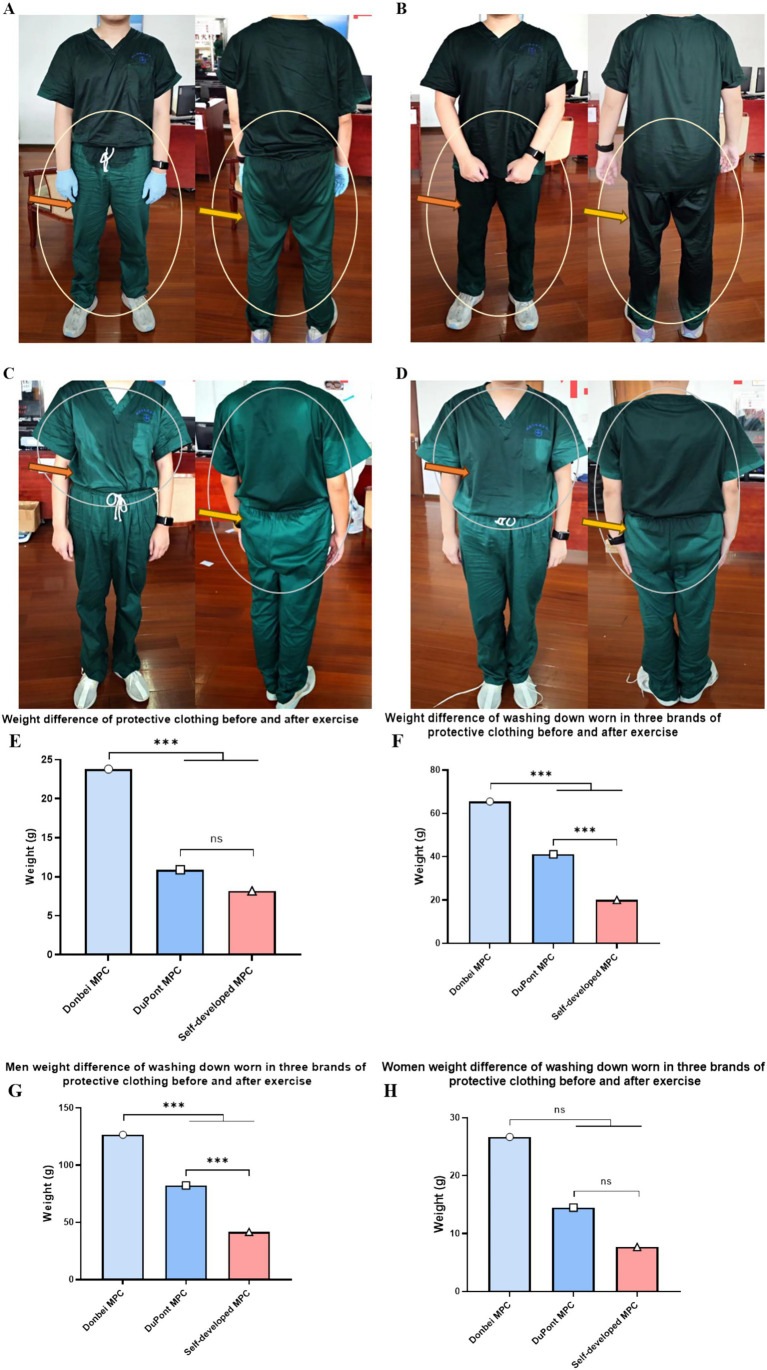
Weight difference between protective clothing and operating clothes before and after exercise. **(A)** and **(B)** respectively refer to the sweating condition of the same volunteer after exercise wearing self-developed MPC and Donbei MPC; **(C)** and **(D)** respectively refer to the sweating condition of the same volunteer after exercise wearing self-developed MPC and DuPont MPC; **(E)** Weight increase of different types of MPC before and after exercise; **(F)** The weight gain of the operating clothes worn by the subjects after exercising with different MPC; **(G)** Weight gain from operating clothes worn by male volunteers before and after exercise; **(H)** Weight gain from operating clothes worn by female volunteers before and after exercise. (Male *N* = 70, Female *N* = 78, *N* = 148, *P < 0.05, **P < 0.01, ***P < 0.001) (two-way ANOVA with *post-hoc* tests).

The same volunteer wore three different types of MPC, which were weighed and analyzed before and after each exercise. After removing outliers from the data, the weights of 139 cases of Dongbei, 138 cases of DuPont, and 148 cases of self-developed medical protective clothing were analyzed. It was observed that the increase in weight for both the DuPont and self-developed groups was comparatively lower than that in the Dongbei group (Figure 4E).

After exercise, the weight gain of the Dongbei group was the highest, whereas that of the self-developed group was the lowest. Statistically significant differences were observed among the three groups (Figures 4A–H).

### Physiological index testing

#### Heart rate

**Figure 5:**
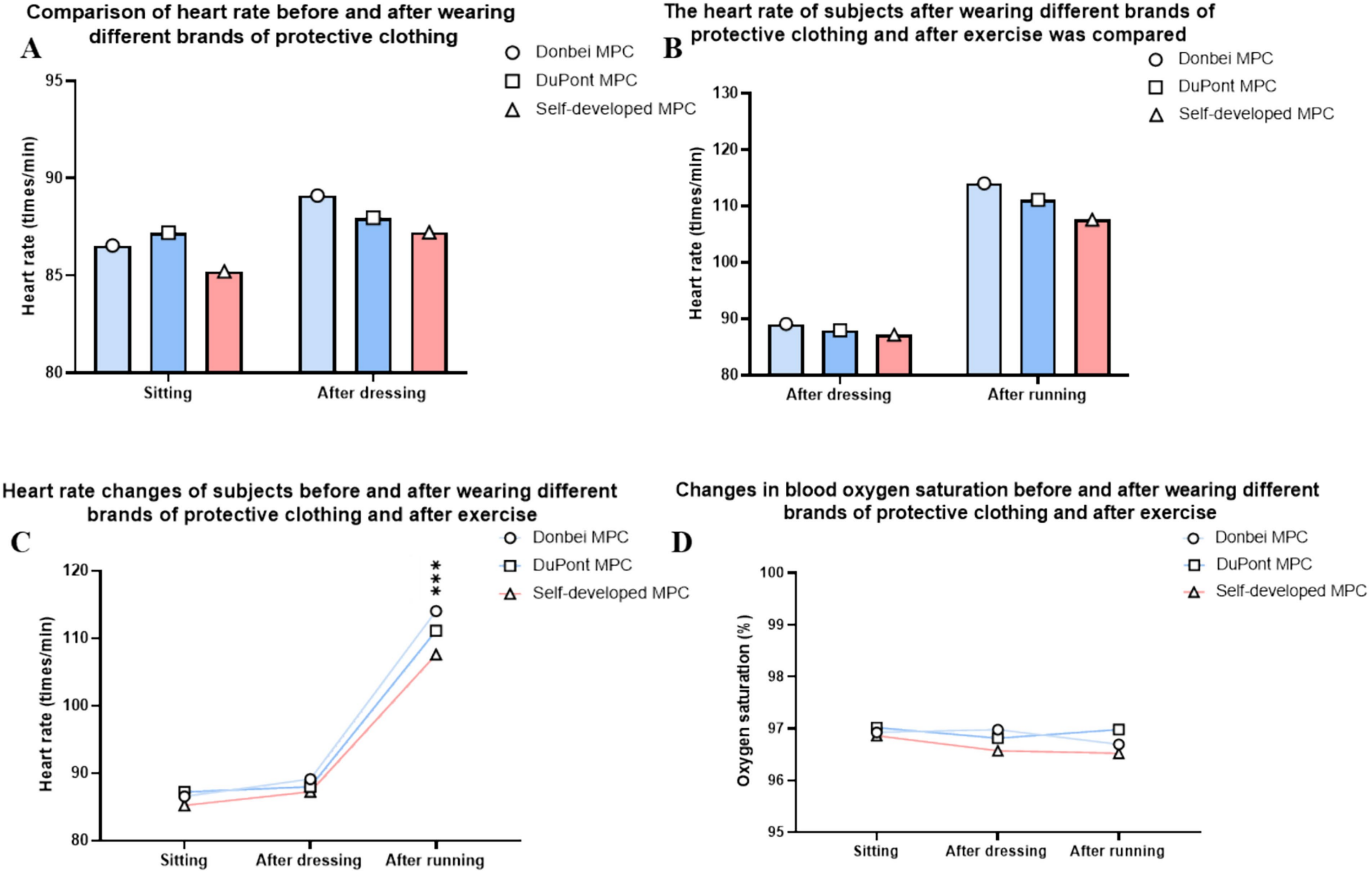
Physical Indicator. **(A)** Resting heart rate comparison after wearing MPC; **(B)** Heart rate changes before and after exercise; **(C)** Heart rate change trend in different states; **(D)** Blood oxygen saturation in different states. (ns *P* > 0.05, *p ≤ 0.05, **p ≤ 0.01,***p ≤ 0.001) (two-way ANOVA with *post-hoc* tests).

The heart rate of each volunteer was measured before donning the MPC, 20 min after donning, and 30 min after running the MPC (26–29). The results showed that there was no significant difference between the groups at sitting compared to before donning (Figure 5A). After sitting and exercising, the increase in heart rate was fastest in the Dongbei group, middle in the DuPont group, and lowest in the self-developed group (Figure 5B). There was a statistically significant difference between the increase in heart rate of the three groups wearing MPC, and the heart rate index of the three groups showed a significant rising trend before and after exercise (Figure 5C).

#### Body temperature, blood sugar, and blood pressure analysis

The body temperature (26), blood sugar, and blood pressure of each volunteer were measured before donning the MPC, 20 min after donning, and 30 min after running the MPC. The results indicated that there was no significant difference in the above indexes between the three protective suits and the wearers at different wearing stages.

#### Oxyhemoglobin saturation

The blood oxygen saturation of each volunteer was measured before donning the MPC, 20 min after donning, and 30 min after running the MPC. The results indicated that the three types of MPC had little effect on the oxyhemoglobin saturation of volunteers, and there was no significant difference between the different wearing stages (Figure 5D).

#### Skin temperature

**Figure 6:**
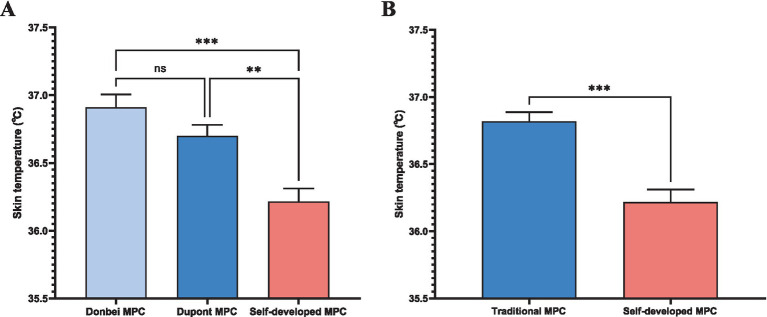
Skin temperature. **(A)** Skin temperature comparison among Donbei MPC, Dupont MPC, and Self-developed MPC. Data are presented as mean ± SD. Statistical significance: ns (not significant), (ns *P* > 0.05, *p ≤ 0.05, **p ≤ 0.01, ***p ≤ 0.001) (two-way ANOVA with post-hoc tests); **(B)** Skin temperature comparison between the average of Donbei and Dupont MPCs (Traditional MPCs) and self - developed MPC. Data are presented as mean ± SD. (***p ≤ 0.001) (unpaired *t* - test).

Figure 6A compares skin temperatures of Donbei MPC, Dupont MPC, and the self - developed MPC. Donbei MPC shows a mean skin temperature of 36.9 ± 0.2°C, Dupont MPC reaches 36.7 ± 0.2°C, while the self - developed MPC records a lower 36.3 ± 0.2°C. Statistical tests indicate no significant difference (ns, *p* > 0.05) between Donbei and Dupont MPCs. However, the self - developed MPC exhibits significantly lower temperatures (*p* < 0.01**) versus Dupont MPC and (*p* < 0.001**) versus Donbei MPC. Figure 6B groups Donbei and Dupont as “Traditional MPC” (mean = 36.8 ± 0.2°C) against the self - developed MPC. The self - developed MPC has a notably lower temperature (36.3 ± 0.2°C), with (*p* < 0.001***), underscoring its superior thermal performance in cutting heat - related risks and boosting healthcare workers’ wear compliance.

### Subjective test

#### Objective evaluation

The Wechsler Adult Intelligence-memory Scale V4.0 was used to comprehensively evaluate the effects of wearing different MPCs on subjects’ short-term working memory and other abilities after a certain period of exercise. The results showed that both the reaction time and working memory of the subjects before and after wearing MPC were not significantly different.

#### Subjective evaluation

**Figure 7:**
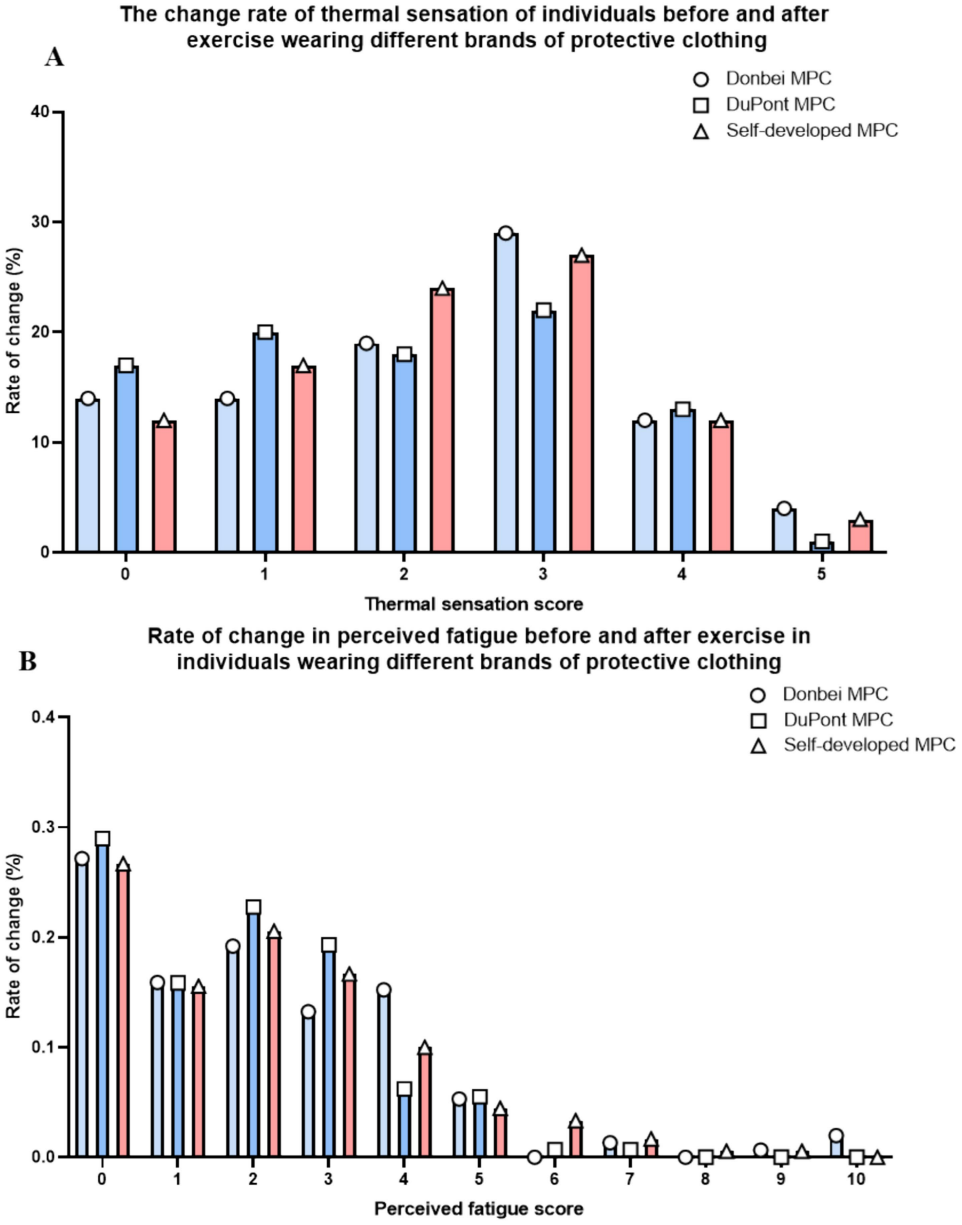
Subjective evaluation. **(A)** Percentage increase of the personal thermal state sensory rating scale of all subjects at different exercise stages; **(B)** The percentage increase of subjective sensory fatigue rating scale of all subjects in different exercise stages.

The subjects were asked to evaluate different MPCs before and after exercise using the subjective fatigue scale, personal thermal Sensation Rating scale (30, 31), for specific scales, please refer to Supplementary Tables S1, S2. The results showed that in terms of subjective fatigue and thermal comfort, the change in the subjective evaluation of each MPC was consistent with the trend of change. There were no significant difference in the increase degree of fatigue and thermal sensation (Figures 7A,B).

Subjective evaluations of the three types of protective clothing were conducted based on ISO standard rating scales (Supplementary Tables S1, S2). However, as the evaluations were performed shortly after intense exercise when subjects’ bodies had not fully recovered, the assessments failed to fully reflect objective experiences related to sweating volume and breathability. This led to discrepancies between subjective perceptions and actual breathability test results.

Furthermore, no significant differences were observed in subjects’ responses and judgments during the subjective evaluations. Analyses combined with results from the Wechsler Adult Intelligence Scale suggested that the experimental workload, predominantly consisting of treadmill running, inadequately simulated the complex cognitive demands and stressful atmosphere of real - world medical rescue scenarios.

Consequently, this limitation contributed to the lack of significant differences in subjective test outcomes.

## Discussion

In 2030, it’s estimated that around 80 million people will be working in the global healthcare industry (32). During the pandemic of infectious diseases, wearing protective clothing for a long time can cause problems related to air permeability (7), and in severe cases, even suffocation. Besides ensuring protective safety, the comfort of Medical Protective Clothing (MPC) has become a major concern. Based on the split - type disposable medical protective clothing, we developed a new integrated disposable medical protective clothing by improving the design and materials.

We have made significant changes to the traditional open - style protective clothing. Instead of the old combination of a mask, goggles, and separate clothing, we have adopted an integrated, one - piece sealing design. We’ve enlarged the eye window and enhanced the facial protection. Through ultrasonic seamless welding between the fabric and the mask, we have achieved better mouth and nose protection. This sealing structure can effectively prevent microorganisms (33), viruses, and bacteria from entering through the joint between the mask and the clothing.

In terms of convenience, our self - developed protective clothing has a much shorter donning and doffing time compared to the two commonly used MPCs in clinical settings, DuPont and Dongbei. The integrated design also eliminates the cumbersome wearing steps of the split - type protective clothing. This convenience can significantly boost the daily work efficiency of medical staff. Second, the integrated protective clothing offers more complete protective and sealing performances than the split - type, reducing the risks of negligence caused by fatigue during wearing and taking off (34). This can enhance the potential protection efficiency and lower the infection rate in the working environment of medical workers. Third, the integrated protective clothing has far fewer binding parts around the mouth compared to the split - type, which helps reduce skin scratches and ulcers caused by long - term wearing. Finally, the one-piece protective suit reduced the frequent use of disposable masks, alleviating the environmental problems caused by the massive use of disposable masks during the epidemic (35, 36). All in all, considering the actual work and protective needs of medical personnel, the integrated one - piece protective clothing is a superior choice.

By comparing the weights of the protective clothing and operating clothes before and after exercise, we found that our self - developed protective clothing can greatly reduce the amount of sweat inside, and the improved moisture permeability significantly enhances work comfort. The comfort of disposable medical protective clothing is mainly linked to its thermal comfort, which depends on factors like the material, wearing duration, activity level while wearing, and the use scenario. Insulated protective clothing offers the highest level of safety, but it’s extremely uncomfortable to wear due to its lack of air and moisture permeability (10). Currently, most disposable medical textiles, such as surgical caps, gowns, and masks, are mainly made of non - woven materials (37). These non - woven materials, usually made of polypropylene with a spunbond - melt - blown - spunbond (SMS) structure, can be produced quickly and inexpensively (38, 39). Nevertheless, the filtration efficiency of SMS on solid particles is insufficient, only about 60%. Thus, in infectious areas, the simple use of SMS nonwovens to make protective clothing cannot meet the safety protection requirement (40). In the product catalogs of 3 M, DuPont, and other companies, most protective clothing is made of composite film fabric. This fabric, usually made by bonding or hot - rolling a non - porous or microporous film with a woven or non - woven fabric, can block liquids (41, 42) and aerosols, while allowing water vapor to pass through, providing good wearing comfort. In our experiment, we chose a high - performance antistatic polypropylene non - woven fabric - based breathable nanomembrane as the main material for the newly developed protective clothing. We increased the weight of the polypropylene non - woven fabric to 45–50 g/m^2^ and improved its weft strength to meet and exceed the requirements of the GB 19082–2009 (Chinese) standard. We also optimized the membrane thickness and aperture and applied different composite processes like solvent adhesive, hot melt adhesive, and hot - pressing composite to solve the problem of fabric stripping (43). In the end, we selected a polypropylene non - woven fabric - based composite breathable nanomembrane that meets both breathability and moisture permeability requirements. The permeability of the tested fabric reached 23 mm/s or higher, and the moisture permeability reached 6,900 g/(m^2^·24h).

In recent years, the remarkable progress in bionanomaterials has significantly broadened the prospects for future medical protective clothing. Nanomaterials, with their unique physical and chemical properties at the nanoscale, have opened up new possibilities for enhancing the performance of medical protective clothing. For instance, the development of electrostatic spinning nanofiber technology has led to the creation of materials like Surforce® nanofiber membranes. These membranes, composed of countless nanoscale fibers intertwined in a complex network, possess highly interconnected pore channels. This structure enables efficient passage of a large amount of moisture and air, effectively reducing the feeling of stuffiness during long - term use, while simultaneously maintaining excellent resistance against infectious fluids, patient blood secretions, and airborne particles (44, 45). Therefore, there remains substantial room for improvement in the materials of the protective clothing proposed in this paper. Future research could focus on exploring novel materials with enhanced breathability, moisture - wicking properties, and comfort, aiming to minimize the discrepancies between subjective evaluations and objective performance, while also better simulating the actual working conditions of medical staff.

Strengths: The study adopts a rigorous comparative test design, directly comparing the new integrated MPC with mainstream products (DuPont and Dongbei), and comprehensively evaluates its performance through multiple indicators such as physiological tests and subjective evaluations. The new MPC not only improves comfort while ensuring protective performance, but also shortens the donning/doffing time, which has strong practical application value.

Limitations: The age range of the subjects is relatively narrow (25–45 years), which may limit the generalization of the results to medical staff outside this age group. In addition, the experimental scenario (treadmill exercise) can only partially simulate the complex working environment of medical staff, and the actual working conditions involve more diverse movements and longer working hours.

To sum up, the integrated medical protective suit design shortens the donning and doffing time, overcomes the drawbacks of traditional protective wear such as poor air permeability and discomfort, and can somewhat reduce the large fluctuations in heart rate after intense exercise. There were only minor differences in heart rate, blood glucose, blood oxygen, and subjective and objective evaluations among the three MPCs. This could be due to the relatively light task load in the experiment, which did not reach the human tolerance limit, allowing the body to make self - compensatory adjustments. On the other hand, it also shows that our self - developed protective clothing does not cause abnormal physiological indicators, and we can adjust the experimental task design for further testing.

## Conclusion

The integrated medical protective clothing (MPC) developed in this study achieves a balance of protection, comfort, and operability, addressing practical dilemmas faced by healthcare workers during prolonged use in infectious disease scenarios. Its improved thermal comfort, reflected in lower skin temperature and reduced sweating, directly eases physical discomfort during extended shifts, helping maintain work efficiency. The streamlined donning and doffing process shortens operation time, which is particularly beneficial for busy clinical workflows requiring frequent gear changes. With fewer potential leakage points, this MPC enhances protective reliability, reducing the risk of healthcare-associated infections in daily practice. Such a design that integrates functional optimization with user needs provides a feasible solution for upgrading medical protective equipment. In application, it can better support frontline healthcare workers in fulfilling their duties safely and effectively, especially in intensive care settings during infectious disease outbreaks, contributing to more robust on-site infection control.

## Data Availability

The original contributions presented in the study are included in the article/Supplementary material, further inquiries can be directed to the corresponding author/s.
