# Supplementary material for: Improved medical protective clothing comfort for medical staff facing high-intensity infectious diseases
Source: Front Public Health. 2025 Aug 11;13:1643043. doi: 10.3389/fpubh.2025.1643043 (PMC12375584; doi:10.3389/fpubh.2025.1643043)
Supplement: Supplementary file 1 [file Table_1.docx]

**Supplementary Material for**

Improved Medical Protective Clothing Comfort for Medical Staff Facing High intensity Infectious Diseases

Chengteng Jiang *et al.*

Corresponding author: Chuang Nie, liz137@163.com.

Main Corresponding author: Jian-wen Gu, JwenGu@163.com.

**Table S1. Subjective perceived fatigue scale**

| Grade of fatigue | Description of fatigue rating | The degree of fatigue | | Choice |
| --- | --- | --- | --- | --- |
| 0 | Nothing at all |  | |  |
| 0.5 | Very, very weak | | (just noticeable) |  |
| 1 | Very weak |  | |  |
| 2 | Weak | (light) | |  |
| 3 | Moderate |  | |  |
| 4 | Somewhat strong |  | |  |
| 5 | Strong | (heavy) | |  |
| 6 |  |  | |  |
| 7 | Very strong |  | |  |
| 8 |  |  | |  |
| 9 |  |  | |  |
| 10 | Very, very strong | (almost max) | |  |

(Please combine your current fatigue feeling and its quantitative proportional relationship with the maximum fatigue feeling you can withstand, and score your fatigue from 0 to 10 points from weak to strong, please draw √ next to your choice).

**Table S2. Thermal state Sensory Rating Scale**

| Feeling | Grade of feeling | The degree of feeling | Choice |
| --- | --- | --- | --- |
| HOT | +5 | Extremely hot |  |
|  | +4 | Very hot |  |
|  | +3 | Hot |  |
|  | +2 | Warm |  |
|  | +1 | Slightly warm |  |
| INDIFFERENCE | 0 | Neutral |  |
| CLOD | -1 | Slightly cool |  |
|  | -2 | Cool |  |
|  | -3 | Cold |  |
|  | -4 | Very cold |  |
|  | -5 | Extremely cold |  |

(How do you feel now? Please rate your choices on a scale of 0 for feeling normal: "extremely cold" to -5 and "extremely hot" to +5. Tick √ next to your choices).
